# *RIN2* reveals a novel role in regulating proliferation and early adipogenesis of chicken preadipocytes

**DOI:** 10.3389/fvets.2026.1770852

**Published:** 2026-04-27

**Authors:** Wujian Lin, Tuanhui Ren, Xiuxian Yang, Shizi He, Wangyu Li, Wen Luo, Guohui Zhu, Xiquan Zhang

**Affiliations:** 1 Guangdong Provincial Key Laboratory for the Development Biology and Environmental Adaptation of Agricultural Organisms, College of Life Sciences, South China Agricultural University, Guangzhou, China; 2 State Key Laboratory of Swine and Poultry Breeding Industry, and Guangdong Laboratory for Lingnan Modern Agriculture, Guangzhou, China; 3 Department of Animal Genetics, Breeding and Reproduction, College of Animal Science, South China Agricultural University, Guangzhou, Guangdong, China; 4 Shenzhen Branch, Guangdong Laboratory for Lingnan Modern Agriculture, Key Laboratory of Livestock and Poultry Multi-omics of MARA, Agricultural Genomics Institute at Shenzhen, Chinese Academy of Agricultural Sciences, Shenzhen, China; 5 Faculty of Animal Science and Technology, Yunnan Agricultural University, Kunming, China; 6 Guangdong Provincial Key Laboratory of Agro-Animal Genomics and Molecular Breeding, and Key Lab of Chicken Genetics, Breeding and Reproduction, Ministry of Agriculture and Rural Affair, Guangzhou, Guangdong, China

**Keywords:** adipogenesis, chicken, fat deposition, *in vivo*, RIN2

## Abstract

Adipose tissue accumulation represents a significant challenge to both human metabolic health and agricultural productivity. While Ras and Rab Interactor 2 (*RIN2*) has been characterized as a Rab5 effector protein, its precise role in adipogenesis remains poorly defined. Here, we identified *RIN2* as a novel critical regulator of adipogenesis in chicken. We found that *RIN2* was widely expressed across various tissues and dynamic expression patterns during adipocyte differentiation. Through comprehensive functional analyses in both ICP-1 cells and primary preadipocytes, we identified that *RIN2* overexpression enhances cellular proliferation, cell cycle progression, and adipogenic differentiation, whereas *RIN2* knockdown produced contrasting inhibitory effects. Transcriptomic profiling uncovered that *RIN2* functions through modulation of the cholesterol metabolic pathway and promotes fatty acid metabolism. Most notably, *in vivo* knockdown of *RIN2* specifically reduced abdominal fat deposition without compromising other carcass characteristics. In summary, our findings identify *RIN2* as a key regulator of fat accumulation and highlight its potential as a genetic target for improving poultry carcass composition.

## 1 Introduction

Adipose tissue is an essential endocrine organ releasing hormones and other cytokines that can deeply affect our health (1–4). It contains many components such as immune cell, preadipocyte, adipocyte, connective tissue matrix, nerve tissue, stromovascular cell and stem cell (3). Abdominal fat and subcutaneous adipose tissue are major types of adipose tissue but researchers imply that abdominal fat cell is more biologically active (5). Among these, abdominal fat deposition is of particular concern as it's a key factor in various health problems (6, 7). Chicken has a long history as a valuable model research organism, a number of traits make them a viable model for studies of obesity, adipose biology and insulin resistance (8, 9). Broiler chickens are naturally hyperglycemic and insulin resistant and rapidly accumulate adipose tissue due to intensive genetic selection, making them can be used as a good biomedical model to study obesity or obesity-related disease. Meanwhile, Genetic selection for meat production of broiler chickens concomitantly causes excessive abdominal fat deposition, accompanied by several adverse effects, such as the reduction of carcass production and meat quality (10). Therefore, understanding the mechanisms underlying abdominal fat deposition is crucial, not only for its impact on physiological health but also for optimizing production efficiency and reducing costs.

The Ras and Rab Interactor 2 (*RIN2*) gene belongs to the Ras superfamily and interacts with Ras and Rab small GTPases, which are essential for intracellular signaling and membrane trafficking (11). Previous research has shown that *RIN2* syndrome in humans is also called MACS (macrocephaly, alopecia, cutis laxa, and scoliosis) syndrome, a rare hereditary skin disease (12, 13). A genome-wide methylation and transcriptome analysis of sheep's longest muscle indicated that *RIN2* may influence meat quality traits (14). Our previous study revealed that the 61-bp indel in the *RIN2* gene was significantly associated with abdominal fat percentage in chickens and was subject to selection across different breeds (15). However, the specific function of the *RIN2* gene in adipocytes remains poorly understood. In particular, the precise molecular mechanisms and associated signaling pathways through which it regulates fat deposition have not been systematically elucidated.

Through a combination of *in vitro* and *in vivo* analyses, we have determined that the *RIN2* gene enhances fat deposition in chickens by stimulating preadipocyte proliferation and differentiation. The underlying mechanism appears to involve the fatty acid elongation signaling pathway. These results shed light on the molecular regulation of fat accumulation in poultry, thereby contributing to a better understanding of the biological processes related to animal health.

## 2 Materials and methods

### 2.1 Ethics statement and animals

All experimental protocols were approved by the Animal Ethics Committee of Institutional Animal Care and Use Committee of the South China Agricultural University (Guangzhou, China) with approval number SCAU# 2020-C036. Five-week-old (5-female) Mahuang chickens (a Chinese native breed) were used to examine the *RIN2* expression profile in different tissues and high- and low-abdominal fat (abdominal fat, heart, lung, spleen, cerebellum, liver, breast muscle, kidney and leg muscle). The tissue samples were flash-frozen in liquid nitrogen and stored at−80°C. 10–day-old female Mahuang chickens (*n* = 21; 7 per group) were used to assess the *in vivo* effects of *RIN2* on abdominal fat deposition.

### 2.2 Cell culture and transfection

Immortalized chicken preadipocytes (ICP-1) were kindly provided by the Key Laboratory of Chicken Genetics and Breeding, Ministry of Agriculture, Northeast Agricultural University (16). ICP-1 was cultured in medium (DMEM/F12 supplemented with 15% (v/v) fetal bovine serum and 1% penicillin/streptomycin. The cells were cultured in 5% CO_2_ atmosphere at 37 °C. The differentiation of preadipocytes was induced by treating the cells for 48 h with DMEM/F12 medium supplemented with 1% penicillin/streptomycin and 0.05% sodium oleate.

Chicken primary preadipocytes were isolated as previously described (17). In short, the abdominal fat tissue was collected from 14-day-old Mahuang chickens under a sterile atmosphere, and placed in a 4-ml centrifuge tube containing 1 ml DMEM/F12 medium (Invitrogen, Carlsbad, United States), and minced into sections of approximately 1 mm^2^. The growth culture-medium used was normal growth medium (DMEM/F12 supplemented with 15% (v/v) fetal bovine serum (Gibco, Carlsbad, CA, USA) and 1% penicillin/streptomycin (Invitrogen).

All transient transfections were performed using Lipofectamine 3,000 (Invitrogen, Waltham, USA) according to the manufacturer's instructions.

### 2.3 Genomic DNA extraction, CDS regions and bioinformatics analysis

Genomic DNA of Mahuang chickens was extracted from blood using a Blood DNA Kit (Omega, Norcross, America). PCR assay was performed as described before (18). All the primers used in this study were designed using the online tools provided by NCBI and were synthesized by Beijing TsingKe Company (Supplementary Table S2). DNA amplification was performed using these primers, and the products were sent for Sanger sequencing (Beijing TsingKe Company). Finally, the CDS region of the chicken *RIN2* gene was assembled using DNAMAN software. The protein-protein interaction prediction network was analyzed by online software STRING v102 (https://string-db.org/). Phylogenetic tree created using neighbor-joining method by Molecular Evolutionary Genetics Analysis version 7.0 (MEGA7) software.

### 2.4 RNA extraction, cDNA synthesis, and Real-time qPCR

Total RNA was extracted from cells or tissues using TRIzol Reagent (Takara, Dalian, China) according to the manufacturer's instructions. A MonScript™ RTIII All-in-One Mix with dsDNase (Monad, Wuhan, China) was used to synthesize cDNA. The cDNA samples were quantified in a QuantStudio™ 5 qPCR System (ThermoFisher) using ChamQTM Universal SYBR qPCR Master Mix (Vazyme, Nanjing, China) following the manufacturer's protocols. Chicken β*-actin* gene was used as an internal control. Data analyses were performed using the 2^−△△*CT*^ method as described previously (19). All the primers were synthesized by Beijing TsingKe Company (Table S3).

### 2.5 Flow cytometry, EdU, and CCK-8 assays

For cell-cycle analysis, ICP-1 were cultured in 12-well plates, after a 48-h transfection, cells were harvested and fixed in 70% ethanol overnight at−20 °C. With a cell cycle analysis kit (Thermo Fisher Scientific, USA), the cell cycle properties analyzed by a BD Accuri C6 flow cytometer (BD Biosciences, San Diego, CA, USA), and the data were processed using ModFit LT 4.1.7 software (Verity Software House, Topsham, ME, USA). For the EdU assay, ICP-1 were cultured using Cell-Light™ EdU Apollo^®^567 *In Vitro* Imaging Kit (RiboBio, Guangzhou, China) according to the manufacturer's instructions. The images were photographed by an inverted fluorescence microscope (Lecia DMi8, Wetzlar, Germany). For cell-viability assay, ICP-1 were cultured in a 96-well plate and at 60% confluence they were transfected as indicated. The cell viability was detected with TransDetect Cell Counting Kit-8 (TransGen, Beijing, China). Ten μl/well CCK-8-solution was added and the absorbance was measured at 450 nm after incubation for 1 h at 37°C. The CCK-8 assay was performed each day for 5 days.

### 2.6 Western blot analysis

The cells were cultured in 6-well plates, transfected using Lipofectamine 3,000 (Invitrogen, Waltham, USA). Total protein was quantified using the Pierce™ BCA Protein Assay Kit (ThermoFisher, Waltham, USA). Protein lysates were separated by SDS-polyacrylamide gel electrophoresis (Beyotime, China) and transferred onto nitrocellulose membranes (Whatman, Maidstone, UK), and then probed with antibodies following standard procedures. The following antibodies were used: Rabbit Anti-PPAR gamma Polyclonal Antibody (bs-0530R, Bioss, China, 1:1,000), Rabbit Anti-CEBP alpha Polyclonal Antibody (bs-24540R, Bioss, China, 1:1,000), Rabbit Anti-CEBP beta Polyclonal Antibody (bs-1396R, Bioss, China, 1:1,000), Rabbit Anti-Cyclin D2 Polyclonal Antibody (AF5410, Affinity, China, 1:1,000), Rabbit Anti-CDKN1B/p27 KIP 1 Polyclonal Antibody (bs-0742R, Bioss, China, 1:1,000), β-Actin Mouse Monoclonal Antibody (AF0003, Beyotime, China,1:10,000), and HRP-labeled Goat Anti-Rabbit IgG H+L (A0208, Beyotime, China, 1:10,000) were used as a secondary antibody.

### 2.7 Oil-Red-O staining

Cells were cultured in 6-well plates till reaching the confluence of 80% and incubated at 37 °C in a 5% carbon dioxide incubator. The cell layers were fixed and stained with Oil-Red-O staining kit according to the manufacturer's instruction (Sangon, Shanghai, China). Images were captured using a Leica DM2000 LED microscope (Leica, Wetzlar, Germany). For the quantification of Oil-Red-O staining, the stain was extracted in 500μl 100% isopropanol (Aladdin, Shanghai, China) and 100μl was used to measure Oil-Red-O stain in a 96-well plate at OD510nm. 100% isopropanol was used as blank group.

### 2.8 RNA sequencing

For RNA sequencing analysis, ICP-1 were cultured in 12-well plates. When the cells reached 70–80% confluence, they were transfected with plasmid DNA [pEGFP-(Exp)-RIN2 vs. pEGFP-NC]. Then, the RNA samples were sent to Lianchuan Bioinformation Technology Co., Ltd. (Hangzhou, China) for RNA-seq. Paired-end RNA-seq was performed using the Illumina HiSeq 2,000 platform to obtain 101 bp reads. The FPKM (fragments per kilobase of exon per million mapped fragments) values were used to estimate the gene expression levels, and differentially expressed genes (DEGs) were identified using DESeq with the criteria of |log2FC| ≥ 2.0 and padj ≤ 0.05.

### 2.9 Lentivirus production and transduction

The sequence of siRNA-RIN2 (GGAACTAGGCGTGTTTGTT) was given to Hanbio Biotechnology (Hanbio, Shanghai, China) to produce interfering lentivirus. The viral titer of siRNA-RIN2 lentiviruses was 5.0 × 10^8^ TU/ml (HBLV-c-RIN2 shRNA1-ZsGreen-PURO). Control lentivirus (siRNA-NC) was also provided by Hanbio Biotechnology, and the viral titer was 5.0 × 10^8^ TU/ml (HBLV-ZsGreen-PURO NC).

### 2.10 Statistical analysis

All quantitative data are presented as mean ± SD. Each experiment was performed with at least three independent biological replicates, and the exact sample size (*n*) for each group is indicated in the corresponding figure legends. Statistical analyses were conducted using GraphPad Prism. For comparisons between two groups, unpaired or paired two-tailed Student's *t* tests were applied as appropriate. For experiments involving more than two groups, one-way ANOVA followed by *post hoc* multiple-comparison tests was used. A value of *P* < 0.05 was considered statistically significant.

## 3 Results

### 3.1 *RIN2* expression, phylogenetic tree and protein-network interaction

**Figure 1:**
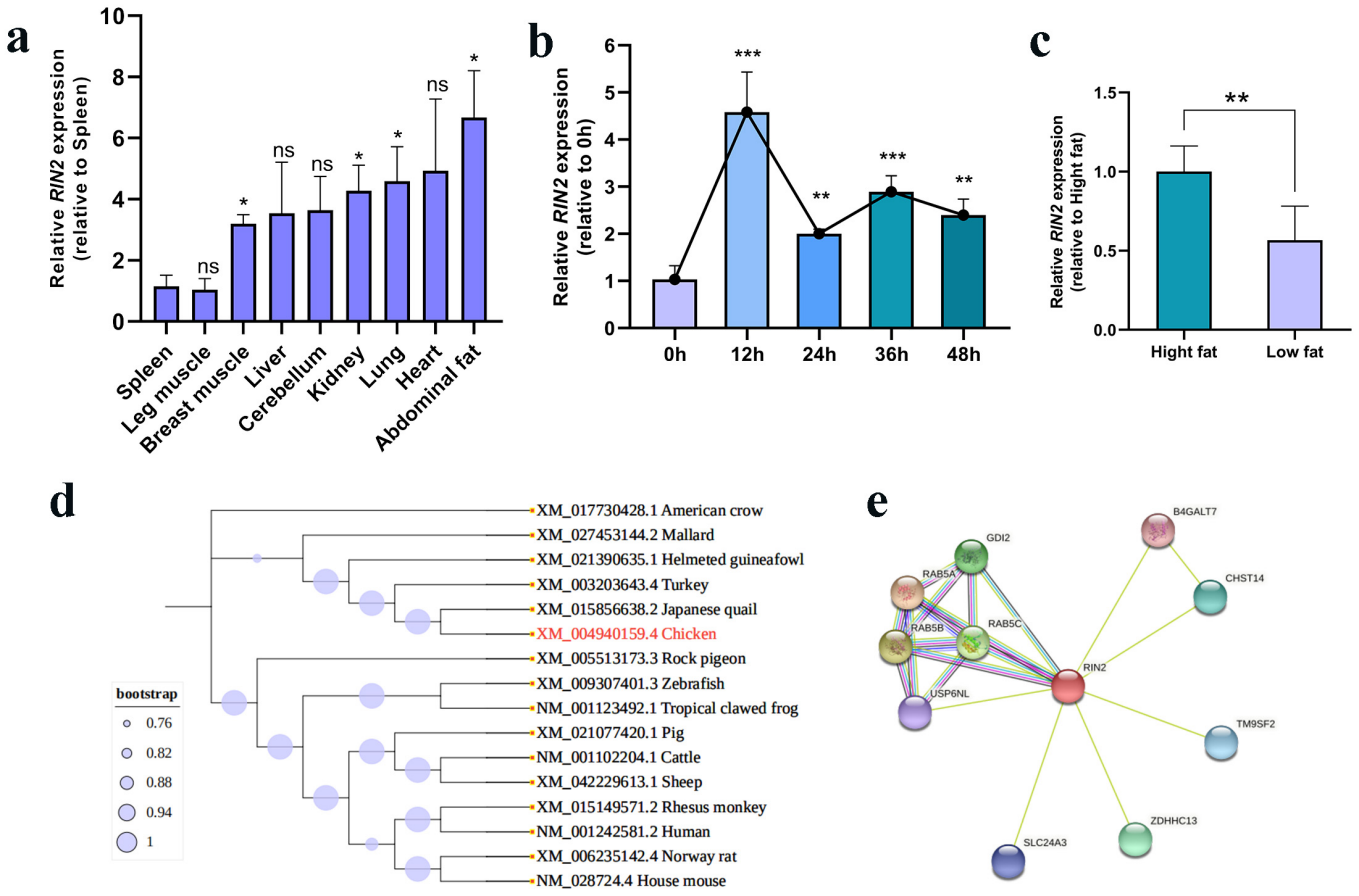
*RIN2* expression profile. **(a)** *RIN2* gene expression in different tissues of Ma Huang chicken, *n* = 5. **(b)** *RIN2* expression profile during adipogenic differentiation of ICP-1, *n* = 3. **(c)** *RIN2* expression profile in high- and low-fat tissues of Ma Huang chicken, *n* = 5. **(d)** Phylogenetic analysis of *RIN2* gene. **(e)** Chicken RIN2 protein interaction network prediction. *p < 0.05, **p < 0.01, ***p < 0.001, ns indicates no significant differences.

RT-PCR results showed that *RIN2* was expressed in 9 examined tissues and the highest expression was found in abdominal fat, heart and liver (Figure 1a). We also investigated the expression of *RIN2* during early chicken preadipocyte differentiation. RT-PCR analysis showed that the level of *RIN2* at 0 h was much lower than that of other times, and the level of *RIN2* mRNA decreased significantly after 12 h of induction, and decreased in a time-dependent manner (Figure 1b). Additionally, *RIN2* was differentially expressed in high- and low-abdominal fat chickens (Figure 1c), and the corresponding slaughter performance data were provided in Table S1. These results suggest that *RIN2* gene may function in the development of preadipocytes and adipocytes. Next, the phylogenetic tree of the *RIN2* gene showed that the phylogenetic relationship among birds and vertebrates was relatively close (Figure 1d). Furthermore, The RIN2 protein network showed Rab5a, Rab5b, Rab5c and GDI2 protein might interact with each other (Figure 1e). Among them, Rab5a, Rab5b and Rab5c are three subtypes of Rab5 protein. And based on the DNA sequence (NCBI Accession Number: XM_040667045.2), we successfully designed four pairs of primers for PCR amplification of the chicken *RIN2* gene CDS regions (supplementary Tables S2, S4) and constructed an overexpression vector using this sequence.

### 3.2 *RIN2* facilitates ICP-1 differentiation

**Figure 2:**
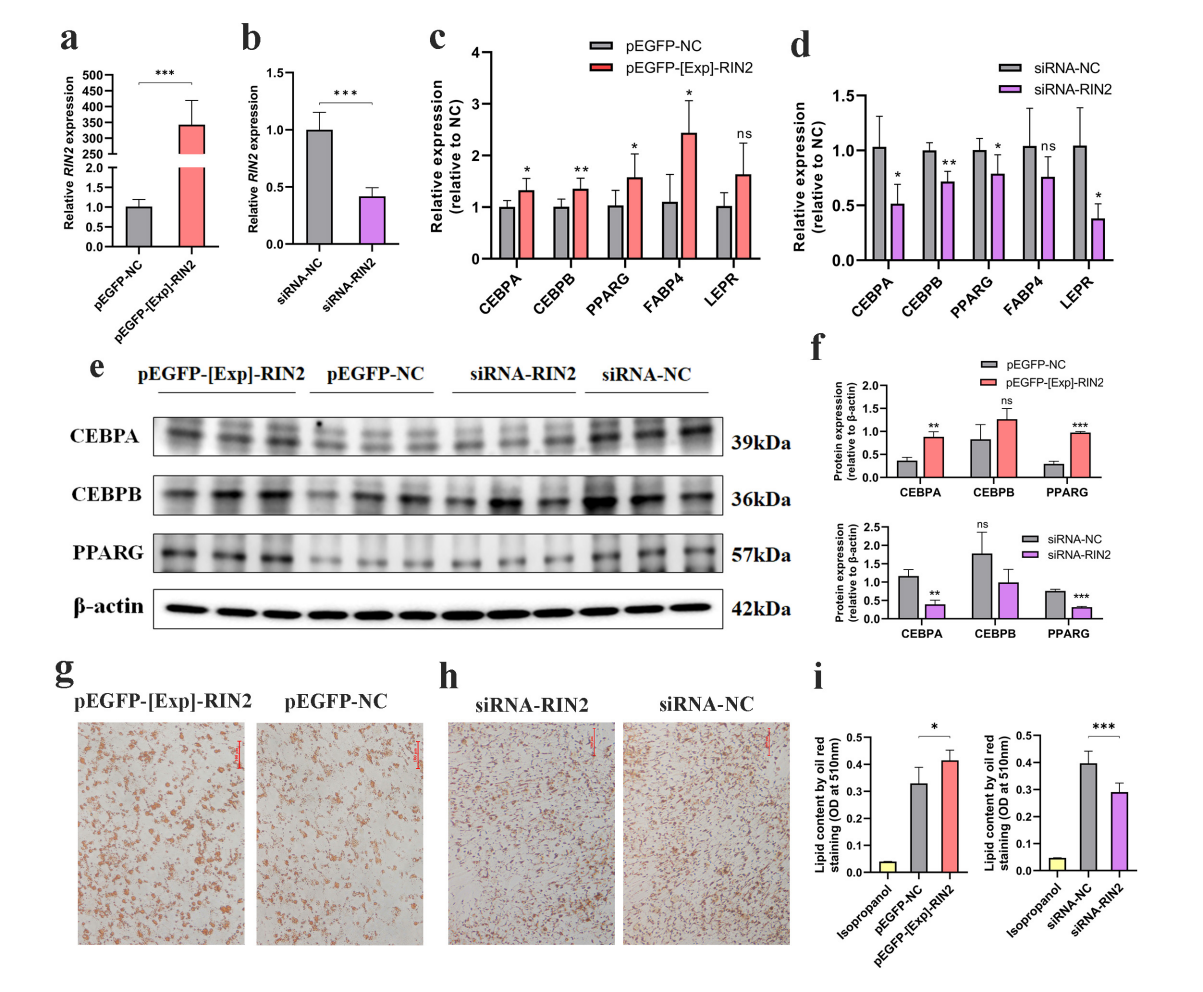
Effects of *RIN2* gene on early differentiation of ICP-1. **(a)** Efficiency of *RIN2* gene overexpression in ICP-1. **(b)** Efficiency of *RIN2* gene knockdown in ICP-1. Effect of *RIN2* overexpression **(c)** and knockdown **(d)** on mRNA expression of adipogenic marker genes. **(e–f)** Effect of *RIN2* overexpression and knockdown on protein expression of adipogenic marker genes. Oil-Red-O staining images of *RIN2* overexpressing **(g)** and knockdown **(h)**. **(i)** Lipid droplet content measured by Oil-Red-O staining after *RIN2* overexpression and knockdown, 100% isopropanol as blank control (IPA-NC). All data are presented as mean ± SD (*n* = 3), *p < 0.05, **p < 0.01, ***p < 0.001, ns indicates no significant differences. ns indicates no significant difference.

We performed *RIN2* overexpression and knockdown experiments in ICP-1 differentiation and their efficiency was detected (Figures 2a, 2b). Furthermore, we evaluate the effects of *RIN2* on the expressions of general adipose markers during the early stage of ICP-1 differentiation. The mRNA levels of *CEBPA, CEBPB, PPARG, FABP* and *LEPR* were detected by qRT-PCR. Additionally, the protein expression levels of CEBPA, CEBPB and PPARG were detected by western blotting after *RIN2* overexpression and knockdown. Results indicated that *RIN2* overexpression could significantly increase the upregulation of general adipose markers (Figures 2c–2f). Conversely, general adipose markers were decrease after *RIN2* knockdown (Figures 2d–2f). Similarly, the results showed that *RIN2* overexpression significantly increased the lipid droplet formation, as judged by Oil-Red-O staining (Figures 2g, 2i). Conversely, *RIN2* knockdown decreased lipid droplet formation (Figures 2h, 2j). Thus, these results suggest that *RIN2* can promote ICP-1 differentiation, a major process in abdominal fat deposition.

### 3.3 *RIN2* facilitates ICP-1 proliferation and induces cell cycle vitalize

**Figure 3:**
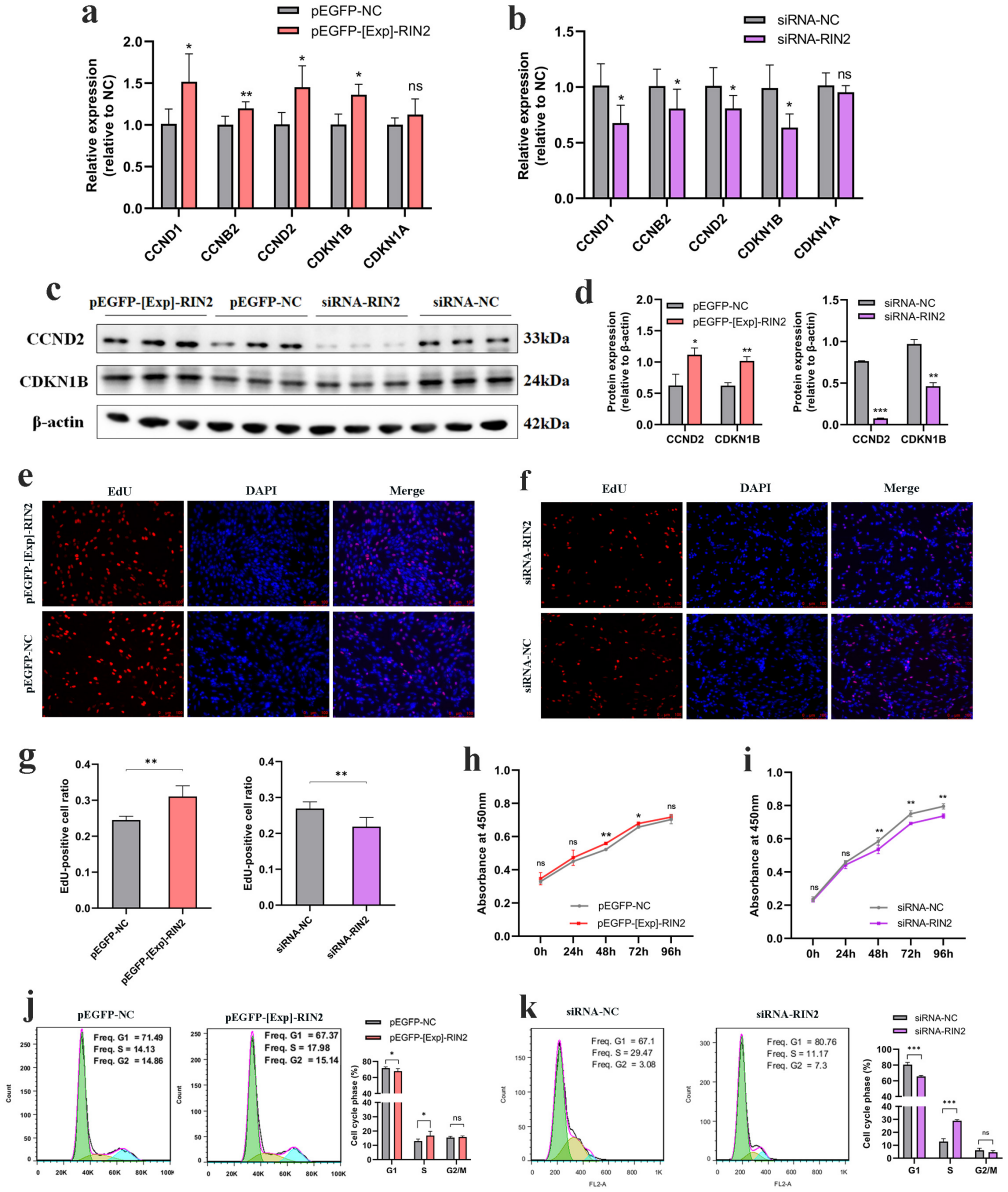
Effects of *RIN2* gene on proliferation of ICP-1. **(a)** Effect of *RIN2* overexpression on mRNA expression of proliferation marker genes. **(b)** Effect of *RIN2* knockdown on mRNA expression of proliferation marker genes. **(c–d)** Effect of *RIN2* overexpression and knockdown on protein expression of proliferation marker genes. Representative fluorescence images of EdU proliferation assay following *RIN2* overexpression **(e)** and knockdown **(f)**. **(g)** Quantification of EdU-positive cells following *RIN2* overexpression and knockdown. **(h)** *RIN2* overexpression alters ICP-1 cell viability in CCK-8 assay. **(i)** *RIN2* knockdown alters ICP-1 cell viability in CCK-8 assay. Cell cycle analysis by flow cytometry following *RIN2* overexpression **(j)** and knockdown **(k)**. All data are presented as mean ± SD (*n* = 3), *p < 0.05, **p < 0.01, ***p < 0.001, ns indicates no significant differences.

We detected the expression levels of some genes involved in cell-cycle regulation after *RIN2* overexpression or knockdown. Results showed that overexpression of *RIN2* significantly increased *CCND2* mRNA and protein level (Figures 3a, 3c, 3d), whereas knockdown *RIN2* expression significantly decreased *CCND2* mRNA and protein level (Figures 3b–3d). Meanwhile, the expression of other cell-cycle related markers, such as *CCND1, CCNB2* and *CDKN1B* were significantly altered but *CDKN1A* remained unaffected (Figures 3a, 3b). The coordinated expression of *CCND2* and *CDKN1B* suggests that cell cycle regulation is likely driven by the integrated effects of multiple regulators rather than a single gene. Furthermore, CCK-8 and EdU assays were conducted to explore the potential role of *RIN2* during preadipocyte proliferation. Results showed that *RIN2* overexpression significantly increased the cell viability or the proliferation of ICP-1 (Figures 3e, 3g, 3h). Conversely, proliferation was decreased after *RIN2* knockdown (Figures 3f, 3g, 3i). Flow cytometry analysis showed that *RIN2* overexpression promoted cell cycle transition from G1 to S phase (Figure 3j), while *RIN2* knockdown arrested the cell cycle in the G1 phase, resulting in delayed cell cycle progression (Figure 3k). Thus, these results suggest that *RIN2* can promote ICP-1 proliferation.

### 3.4 *RIN2* facilitates chicken primary preadipocyte differentiation

**Figure 4:**
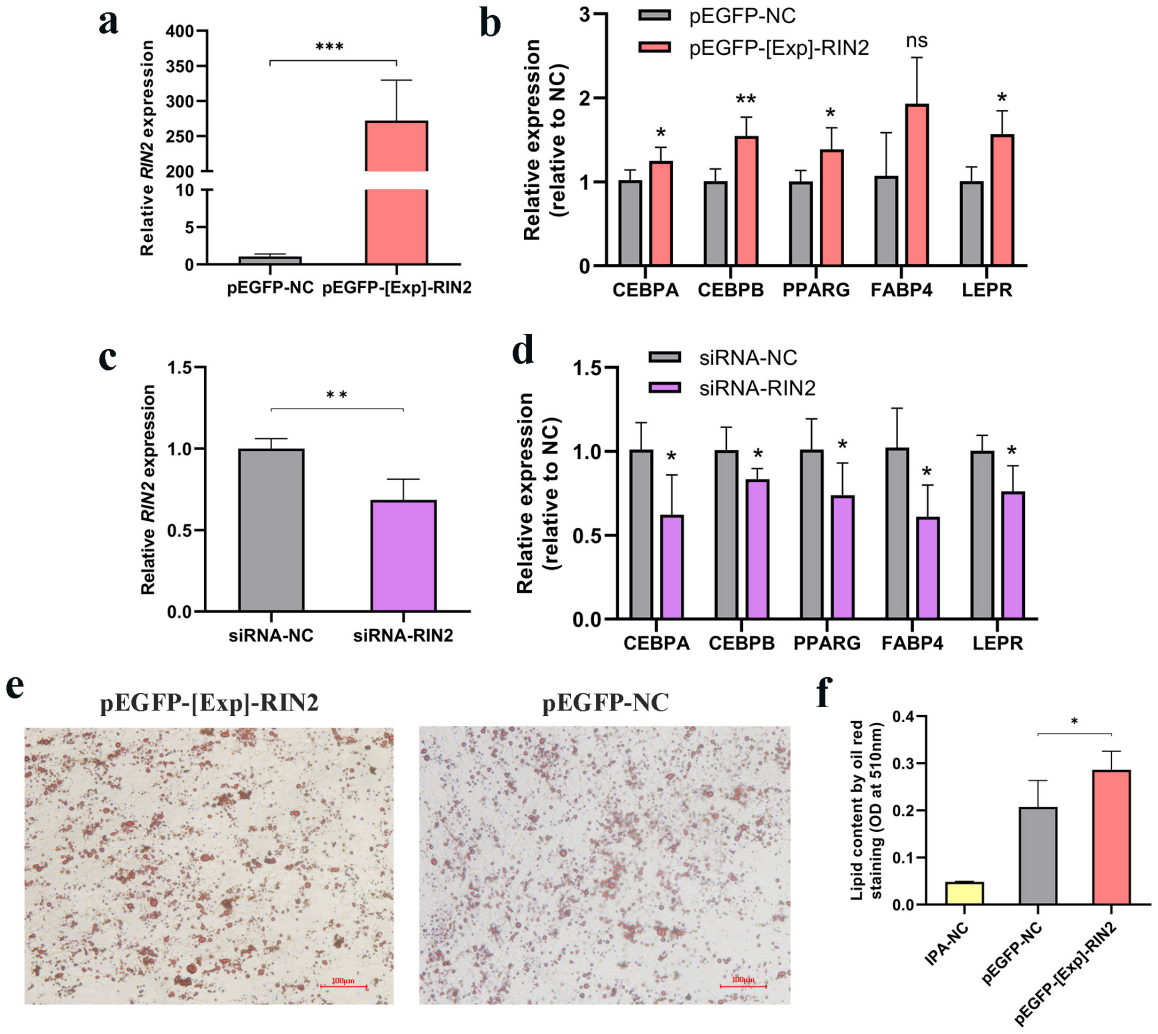
Effect of *RIN2* on early differentiation of primary preadipocytes. **(a)** Efficiency of *RIN2* gene overexpression in primary preadipocytes. **(b)** Effect of *RIN2* overexpression on mRNA expression of adipogenic marker genes. **(c)** Efficiency of *RIN2* gene knockdown in primary preadipocytes. **(d)** Effect of *RIN2* knockdown on mRNA expression of adipogenic marker genes. **(e)** Oil-Red-O staining images of *RIN2* overexpressing. **(f)** Lipid droplet content measured by Oil-Red-O staining after *RIN2* overexpression, 100% isopropanol as blank control (IPA-NC). All data are presented as mean ± SD (*n* = 3), *p < 0.05, **p < 0.01, ***p < 0.001, ns indicates no significant differences.

We explored whether *RIN2* might play a role in the early stage of preadipocyte differentiation. *RIN2* overexpression and knockdown experiments were conducted in chicken primary preadipocytes, and their efficiency was evaluated by qRT-PCR (Figures 4a, c). Next, we evaluate the effects of *RIN2* on the expressions of general adipose markers during the early stage of primary preadipocyte differentiation. The mRNA levels of *PPARG, CEBPA, CEBPB, FABP* and *LEPR* were detected by qRT-PCR after *RIN2* overexpression (Figure 4b) or knockdown (Figure 4d). Results indicated that *RIN2* overexpression could significantly increase the upregulation of general adipose markers. Conversely, general adipose markers were decrease after *RIN2* knockdown. In addition, the Oil-Red-O staining test results showed that *RIN2* overexpression significantly increased the lipid droplet formation (Figures 4e, 4f). These results suggest that *RIN2* promote ICP-1 differentiation. Combining these findings with previous results, we conclude that *RIN2* play a similar role in the early differentiation of both chicken primary preadipocytes and ICP-1 cells.

### 3.5 *RIN2* affects the ICP-1 differentiation transcriptome

**Figure 5:**
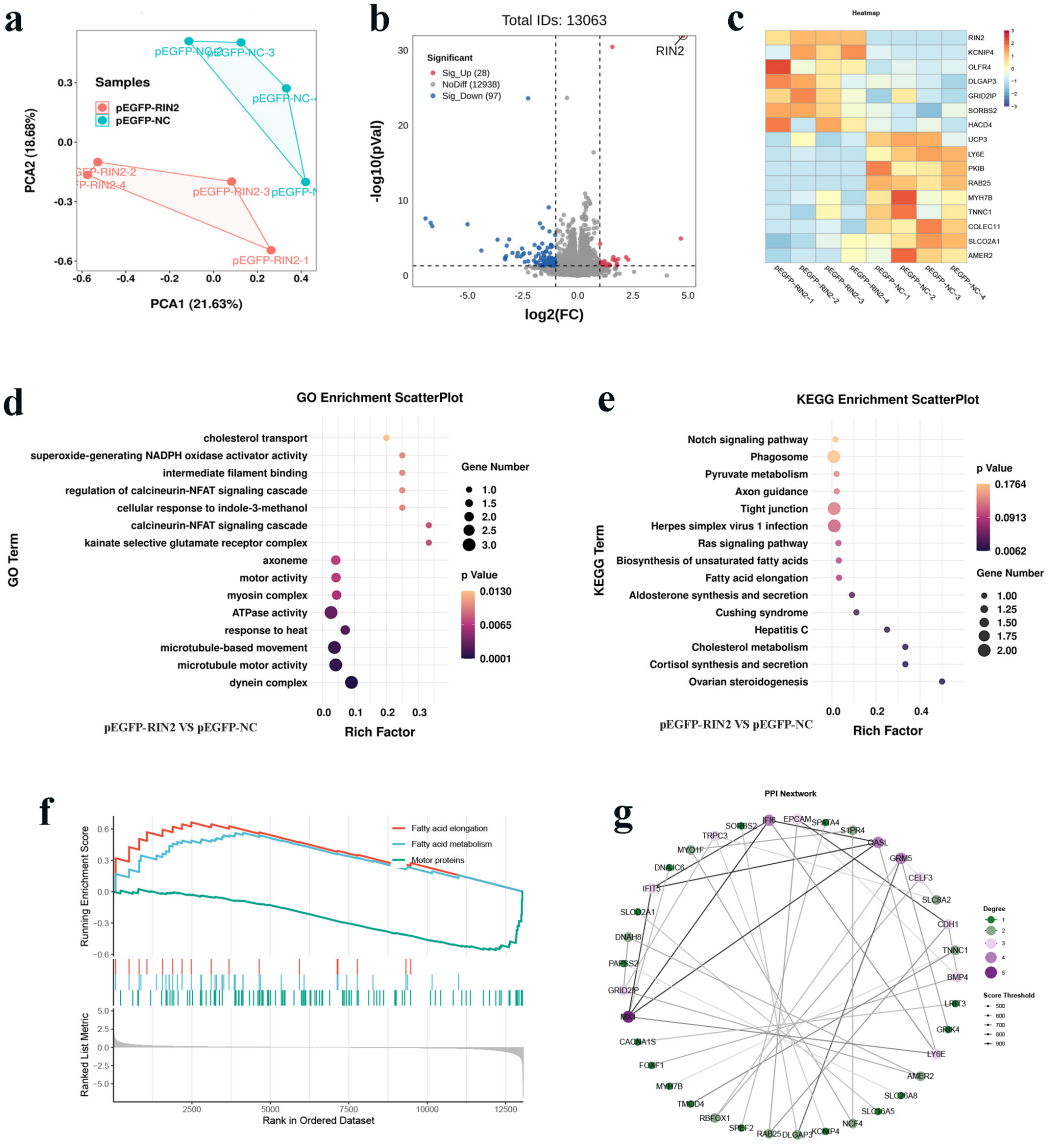
Effect of *RIN2* on the transcriptome of ICP-1. **(a)** PCA of transcriptomic profiles in ICP-1 cells with *RIN2* overexpression during early differentiation. **(b)** Volcano plot of DEGs following *RIN2* overexpression (padj < 0.05, |FoldChange| ≥ 2). **(c)** Heatmap of differentially expressed genes. **(d)** GO analysis of DEGs following *RIN2* overexpression. **(e)** KEGG analysis of DEGs following *RIN2* overexpression. **(f)** GSEA of pathways following *RIN2* overexpression. **(g)** PPI network analysis of DEGs following *RIN2* overexpression.

RNA-Seq technology was used to identify the key differentially expressed genes (DEGs) and signaling pathways after *RIN2* overexpression in ICP-1 differentiation. Results showed that PCA confirmed a high correlation among the biological replicates (Figure 5a). A total of 125 differentially expressed genes (DEGs) were identified in the *RIN2* overexpression group, including 28 upregulated and 97 downregulated genes (Figure 5b). Heat map analysis showed close correlation between biological replicate samples (Figure 5b, c). Next, functional enrichment analysis of DEGs was performed by GO and KEGG pathway analysis. GO functional enrichment analysis showed that the DEGs induced by *RIN2* overexpression were mainly enriched in terms related to cholesterol transport, dynein complex, microtubule motor activity and motor activity (Figure 5d). KEGG pathway analysis revealed that these DEGs were significantly enriched in the Ras signaling pathway, Fatty acid elongation, Cholesterol metabolism, and Pyruvate metabolism pathways (Figure 5e). Importantly, GSEA revealed significant activation of fatty acid elongation and metabolism pathways upon *RIN2* overexpression, whereas motor protein–related functions were markedly suppressed (Figure 5f). PPI network analysis of the dysregulated genes further identified key hub proteins, including EPCAM, S1PR4, and GRM5, suggesting their pivotal roles in the RIN2-mediated regulatory network (Figure 5g).

### 3.6 *RIN2* facilitates abdominal fat deposition in chickens

**Figure 6:**
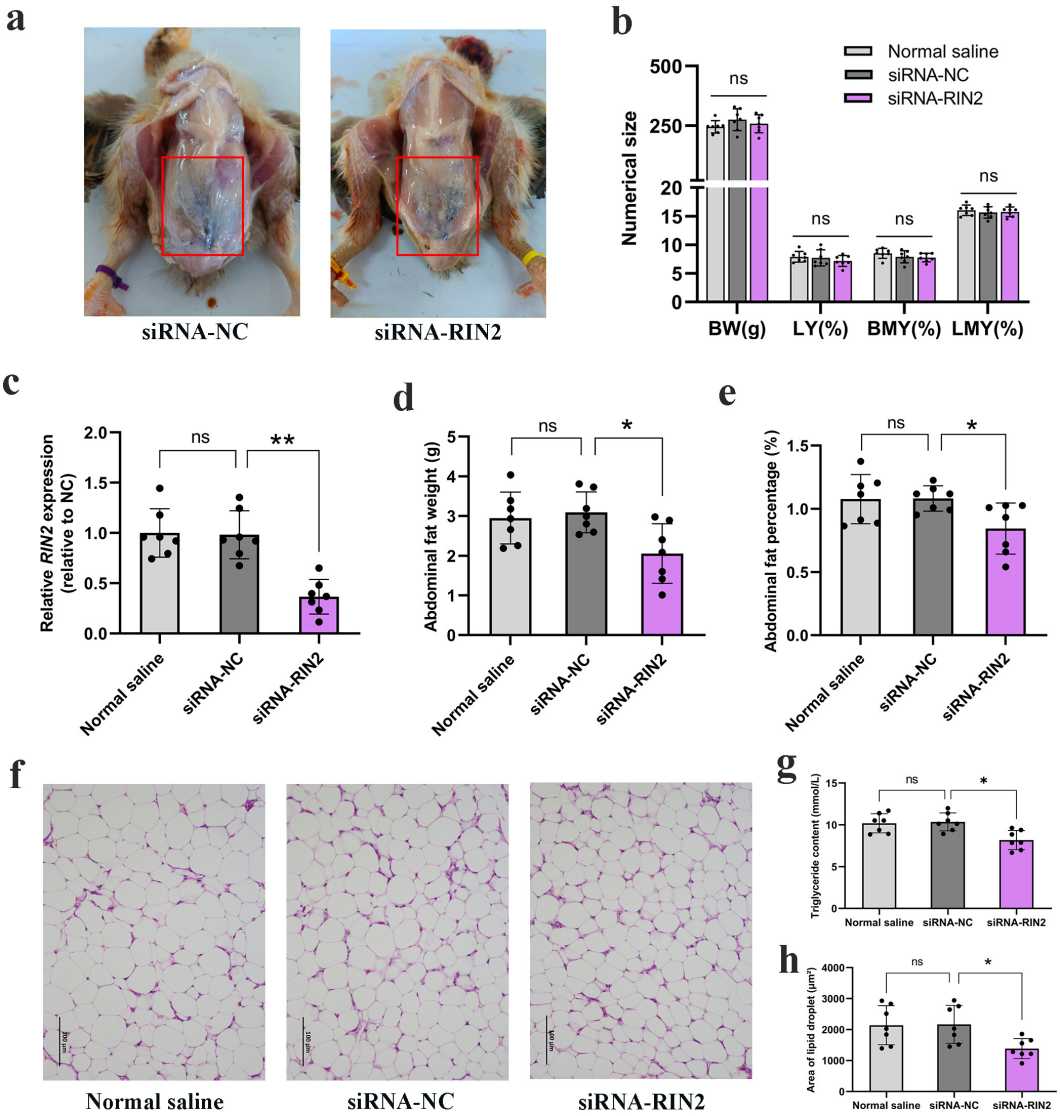
Effect of *RIN2*-interfering lentivirus on abdominal fat deposition in chickens. **(a)** Anatomical images of *RIN2*-interfering lentivirus and control groups. **(b)** Effect of *RIN2*-interfering lentivirus on carcass traits (*n* = 7). BW, Body weight; LY, Liver percentage; BMY, Breast muscle percentage; LMY, Leg muscle percentage. **(c)** Efficiency of *RIN2*-interfering lentivirus. **(d)** Effect of *RIN2*-interfering lentivirus on abdominal fat weight. **(e)** Effect of RIN2-interfering lentivirus on abdominal fat percentage. **(f)** Section images of abdominal adipose tissue following *RIN2* lentiviral interference. **(g)** Effect of *RIN2*-Interfering lentivirus on lipid droplet size. **(h)** Effect of *RIN2*-interfering lentivirus on triglyceride content in abdominal adipose tissue. All data are presented as mean ± SD (*n* = 7), *p < 0.05, ***p < 0.001, ns indicates no significant differences.

To further explore the effect of *RIN2* on chicken abdominal fat deposition *in vivo*, we injected *RIN2* knockdown lentivirus into 10-days-old female chickens at peritoneal cavity. Chickens were divided into 3 groups with 7 replicates each, including the siRNA-RIN2 group, the siRNA-NC group and the Normal saline group. The injection method was multi-point injection in the peritoneal cavity. At 21 days of age, all chickens were slaughtered and carcass traits were measured. Results showed that injection of *RIN2* knockdown lentivirus could significantly reduce the mRNA expression of *RIN2* in abdominal fat (Figures 6a, 6c). Meanwhile, *RIN2* knockdown significantly reduced chicken abdominal fat weight and percentage, while other slaughter performances were unchanged (Figures 6b, 6d–6e). In addition, the lipid droplet area size of abdominal fat was calculated by ImageJ software, and the results showed that *RIN2* knockdown significantly reduced the lipid droplet area size (Figures 6f, 6h). We also analyzed the content of triglycerides in abdominal fat and found that *RIN2* knockdown reduced triglyceride accumulation (Figure 6f, 6g). Overall, *RIN2* exerted a facilitating effect on chicken abdominal fat deposition *in vivo*.

## 4 Discussions

In this study, we systematically revealed that *RIN2* acts as a critical regulator of adipogenesis, orchestrating both the proliferation and differentiation of chicken preadipocytes. Our findings, derived from *in vitro* cellular models and supported by *in vivo* evidence, position *RIN2* as a novel and significant modulator of abdominal fat deposition in chickens.

The widespread tissue expression of *RIN2* suggests it fulfills fundamental cellular functions, a notion supported by its evolutionary conservation across birds and vertebrates. Its marked upregulation during the early phases of adipocyte differentiation in the ICP-1 cell line was particularly notable. This pattern is consistent with a potential role as a licensing factor or a positive regulator that initiates the differentiation program (20, 21). In line with this, our functional assays revealed that *RIN2* overexpression robustly promoted adipogenesis, confirming its positive regulatory function. These results suggested that *RIN2* functions as a licensing factor, where a certain threshold level is required in preadipocytes to prime them for subsequent differentiation. Its differential expression in high- and low-fat chickens further underscored its physiological relevance to fat deposition traits.

Previous studies have shown that mutations in *RIN2* are causative factors of human MACS syndrome (12). In livestock species, *RIN2* has also been implicated in meat quality traits in sheep (14), and genome-wide analyses in Thoroughbred horses suggested that *RIN2* may be associated with racing performance (22). In the present study, our findings identify *RIN2* as a previously unrecognized regulator involved in chicken adipogenesis, suggesting potential functional divergence of this gene during evolution across species.

Adipose tissue development relies critically on the proliferation and differentiation of preadipocytes (23). The pro-adipogenic role of *RIN2* was unequivocally identified through gain- and loss-of-function experiments. The coordinated upregulation of key adipogenic markers (*CEBPA, CEBPB, PPARG*) and the concomitant increase in lipid droplet formation upon *RIN2* overexpression confirm its function in driving the adipogenic transcriptional program. Conversely, *RIN2* knockdown effectively suppressed this process. Beyond differentiation, our data reveal a previously unappreciated role for *RIN2* in promoting cell cycle progression. The upregulation of *CCND2* and the observed G1-to-S phase transition indicate that *RIN2* facilitates preadipocyte proliferation, thereby potentially expanding the pool of cells competent to undergo adipogenesis.

The transcriptomic analysis provides mechanistic insight into *RIN2* function. Enrichment of DEGs in the Ras signaling pathway is consistent with the known role of *RIN2* as a *Rab5* effector. *Rab5* regulates early endocytosis and receptor trafficking (11, 24); therefore, *RIN2*-*Rab5* interaction may influence growth factor and adhesion receptor signaling, leading to downstream transcriptional changes associated with lipid metabolism. The enrichment of cholesterol-related processes suggests that *RIN2* may affect membrane remodeling and adipogenic signaling, as cholesterol is essential for membrane structure and lipid droplet formation (25). In addition, GSEA indicated activation of fatty acid elongation and metabolic pathways, supporting a role for *RIN2* in promoting lipid biosynthesis. Furthermore, the hub genes EPCAM, S1PR4, and GRM5 identified in the PPI network may represent key downstream nodes. EPCAM is associated with epithelial signaling and cell adhesion, which may influence differentiation status (26). S1PR4 participates in sphingolipid signaling and could link membrane lipid dynamics with transcriptional regulation (27). Together, these hub genes suggest that *RIN2* may coordinate membrane trafficking with metabolic reprogramming.

Most importantly, the physiological relevance of our *in vitro* findings was validated *in vivo*. The specific knockdown of *RIN2* in the abdominal cavity significantly reduced abdominal fat pad weight and lipid droplet size without affecting other carcass traits, strongly supporting a direct and specific role for *RIN2* in promoting abdominal fat deposition *in vivo*. This positions *RIN2* as a compelling genetic target for strategies aimed at modulating fat content in poultry breeding.

## 5 Conclusion

Collectively, we have identified *RIN2* as a multifunctional regulator that promotes abdominal fat deposition in chickens by enhancing preadipocyte proliferation and driving adipogenic differentiation. Its effects are likely mediated through the fatty acid elongation and metabolism pathways and critical interactions with proteins like Rab5, ultimately leading to transcriptional reprogramming that favors lipid metabolism and storage. While our study clarifies the overall role of *RIN2*, the precise molecular details of how it interfaces with the Rab5-mediated endocytic machinery to control adipogenic signaling warrant further investigation. Future studies should focus on elucidating these precise molecular cascades and exploring the potential of *RIN2* alleles as markers for selective breeding in poultry.

## Data Availability

Regarding the data deposition, the data presented in the study are deposited in the NCBI repository, with the accession number PRJNA1437011.
