# Supplementary material for: RIN2 reveals a novel role in regulating proliferation and early adipogenesis of chicken preadipocytes
Source: Front Vet Sci. 2026 Apr 27;13:1770852. doi: 10.3389/fvets.2026.1770852 (PMC13158677; doi:10.3389/fvets.2026.1770852)
Supplement: Supplementary file 2 [file Data_Sheet_1.docx]

**Supplementary** **materials**

| **Table S1.** Carcass traits of high- and low-fat Ma Huang chickens | | | | |  |
| --- | --- | --- | --- | --- | --- |
|  |  |  |  |  |  |
|  | number | BW(g) | AFW(g) | AFR(%) |  |
| Hight fat chicken | 1 | 236.15 | 0.49 | 0.207 |  |
|  | 2 | 314.59 | 0.55 | 0.175 |  |
|  | 3 | 292.26 | 0.68 | 0.233 |  |
|  | 4 | 301.57 | 0.91 | 0.302 |  |
|  | 5 | 249.51 | 0.77 | 0.309 |  |
| Low fat chicken | 1 | 306.04 | 2.89 | 0.944 |  |
|  | 2 | 302.72 | 2.42 | 0.799 |  |
|  | 3 | 258.50 | 2.59 | 1.002 |  |
|  | 4 | 280.51 | 2.02 | 0.720 |  |
|  | 5 | 308.52 | 2.13 | 0.690 |  |
| Hight VS Low (*p-value*) | - | 0.504 | 0.001 | 0.001 |  |
|  |  |  |  |  |  |

Note: Body weight (BW); Abdominal fat weight (AFW); Abdominal fat rate (AFR).

**Table S2.** *RIN2* amplified CDS regions primer

| **Gene name** | **Primer sequences (5’-3’)** | **Size**  **(bp)** | **Annealing temperature (℃)** | **Accession number** |
| --- | --- | --- | --- | --- |
| *RIN2-CDS1* | F: AGAACAGTCGCTTGGAGCTG | 742 | 59 | XM_040667045.2 |
|  | R: CTGCAGCAATAGCATGAGGC |  |  |  |
| *RIN2-CDS2* | F: CCTTCAGACAGCGCTAGCAA | 1004 | 60 | XM_040667045.2 |
|  | R: ACTTGGGTCATGAACTGCCG |  |  |  |
| *RIN2-CDS3* | F: GATGCCTGGTGCAGGACTAT | 1175 | 59 | XM_040667045.2 |
|  | R: ACCGTTTCAGCTCCTAAGGC |  |  |  |
| *RIN2-CDS4* | F: ATGTATGCCAGCTGTGTGCT | 314 | 60 | XM_040667045.2 |
|  | R: ACCGTTTCAGCTCCTAAGGC |  |  |  |

**Table S3.** Differentiation and proliferation regulated genes’ primers used for RT-qPCR.

| **Gene name** | **Primer sequences (5’-3’)** | **Size**  **(bp)** | **Annealing temperature (℃)** | **Accession number** |
| --- | --- | --- | --- | --- |
| *RIN2* | F: GCAGATTTGCGAAGTCTAGTAAAGA | 212 | 59 | XM_040667045.2 |
|  | R: AATATCCCAGGAGGCTGTGC |  |  |  |
| *PPAR*γ | F: TCCTTCCCGCTGACCAAA | 227 | 60 | NM_001001460.1 |
|  | R: TCCTGCACTGCCTCCACA |  |  |  |
| C/*EBP*α | F: GACAAGAACAGCAACGAGTACCGC | 195 | 56 | NM_001031459.1 |
|  | R: CCTGAAGATGCCCCGCAGAGT |  |  |  |
| C/*EBP*β | F: GCGGACTGTTTGGCTGCTCT | 220 | 60 | NM_205253.2 |
|  | R: CGGGTGAGGCTGATGTAGGTGT |  |  |  |
| *FABP4* | F: ATGTGCGACCAGTTTGTG | 222 | 56 | NM_204290.1 |
|  | R: TTTGCCATCCCACTTCTG |  |  |  |
| *LEPR* | F: CCAACCCTTCCTTGCTAA | 182 | 60 | NM_204323.1 |
|  | R: GCCTTCAACCCAACATTC |  |  |  |
| *CCND1* | F: CAGAAGTGCGAAGAGGAAGT | 188 | 58 | NM_205381.1 |
|  | R: CTGATGGAGTTGTCGGTGTA |  |  |  |
| *CCNB2* | F: CAGTAAAGGCTACGAAAG | 133 | 58 | NM_001004369.1 |
|  | R: ACATCCATAGGGACAGG |  |  |  |
| *CDKN1B* | F: TCGCTGTGCTGGGCTGAA | 212 | 60 | NM_204256.3 |
|  | R: CAAGGACGAAAGGATGTGGG |  |  |  |
| *CDKN1A* | F: GAGAAGAGTTGTCCACGATAAGCC | 252 | 61 | NM_001396336.1 |
|  | R: CCATTCCAGTCCTCCTCAGTCC |  |  |  |
| *CCND2* | F: AACTTGCTCTACGACGACC | 150 | 55 | NM_001397876.1 |
|  | R: TTCACAGACCTCCAACATC |  |  |  |
| *β-actin* | F: GACTGACCGCGTTACTCCCA | 166 | 61 | NM_205518.2 |
|  | R: CCAACCATCACACCCTGATGTC |  |  |  |

| **Table S4.** Sequence of the synthesized chicken *RIN2* CDS |
| --- |
|  |
| **chicken *RIN2* gene CDS sequence** |
| ATGTCGTGTTTGACGATGAAAGCTCATTGCCGGGACAAAAGAGGAAGTTTCTTTAAGCTCATTGACACGATT GCCTCAGAAATCGGAGAACTGAAACAGGAGATGGTTCAGACAGATGTCACGGTGGAAGATGAGCCTGCAG ATTTGCGAAGTCTAGGAAAGAACATGGATAATGTCTCTCCAGAGAAGAAGGATGTAAAAGGCTGCCCTCGG GACTCTGGATATGACAGCCTATCCAACAAGCTGAGCATATTGGACAAGCTCCTCCATACTCACCCTGTGTGG CTCCAGCTTGGTCTGAATGATACTGAAGCCACAGAAATTCTGCGTGCACAGCCTCCTGGGATATTTTTGGTT AGGAAATCTTCAAAACTGCTGAAGAAGGGCATATCTCTGCGTCTGCCAGGCGACTGTGGGTCCTGCCTGAA GGAATTTGCAATAAAAGAGAGCACATACACGTTTTCCTTAGAGGGGTCTGGAATAAGTTTTGCTGATTTATT CAGGCTCATTGCTTTCTACTGTATTAGTAGAGATGTCCTTCCATTCACCCTGAAGTTGCCTCATGCTATTGCT GCAGCAAAGACAGAAGCTGAACTTGAAGAGATTGCTCAGCTTGGACTGAACTTCTGGAGCTCTCCAGCTAA CAGCAGCCCCTCAGATGCTTCAGCTCCCCATAAGCCTGGGCATTCAGACAGCGCTAGCAAAGACTCGCGTC AGCTCTGCCTTATAAATGGAGTGCATTCTATACGAACCAGAACGCCTTTGGAGCTGGAGTGCAGCCAGACC AACGGAGCGCTGTGTTTCATTAATCCACTCTTCTTAAAAGTGCACAGCCAGGATGTCACTGGAAGTCTGAAA AGGCAGAGCCTGAAATCTCAGGACGTGAATGGCACCGAGAGGCCTCGCTCCCCCCCACCCCGGCCACCACC TCCTTCTATTAATAGCGTCCTCATGAGTCCACAGCTTTCCAGGACTATAAAGCAGGCGAGCGTGCCAGAAAC AGTCAACCATAAGAAAGAGAGAGACTTGGATTTGCTGCAGAGTAAACCAACCCCTATTCCGCCTCCTCGGC TGAAGAAGCAGGCTGTTAGTGCAGAGGTGGAGGGCGGCAGCTCAAAGCCCGCGGCCGTAATTCAACCTGCC TGCAATTCTGTGCGTGTCCCCGCAGCTGCTGACGTTGTAGGTGAAACCCCCCCACCTCAGCCAGCTCCGACG GCTTCCAAGAAGCCTGCAGTTAGCAGCTCTGAGTCACACGTACCCTGGAATGGAGGCAGGCAGAGGCTGAG CGACATGAGCATTTCTACCTCCTCCTCTGACTCGCTGGACTTCGATCGGAGCATGCCGTTGTTTGGCTATGAG GGGGACACTAACAGCAGCCTGGAGGATTTTGAGGGGGAAAGCGACCAAGAGAGCATGGCACCTCCGTTGA AACCCAAGAAGAAGAGAAACAGTTCGTTCGTTCTTCCCAAGATTGTGAAATCCCAGCTACGGAAAGTTAGT GGAGTTTTCAGTTCCTTCATGACCCCTGAAAAGAGAATGATTAAGAAGATTGCAGAGATGTCCCGGGACAA ACGCACTTACTTTGGATGCCTGGTGCAGGACTATATCAGCTTTCTCCAGGAAAACAAGGAGTGCCACGTTTC CAGCACAGATATGCTGCAAACAGTTCGGCAGTTCATGACCCAAGTTAAGAACTATTTGTCCCAAAGCTCTGA ACTTGATCCCCCAATCGAATCGCTGATTCCAGAGGACCAAATAGATGTTGTCCTGGAGAAAGCCATGCATA AGTGCATTCTGAAGCCATTGAAGGGCCACATAGAAGCGATGTTGAAAGAGTTTCATACTGCAGATGGTTCTT GGAAACAGCTAAAGGAAAACCTACAGCTGGTACGGCAGAGGAATCCTCAGGAACTAGGCGTGTTTGTTCCA ACACCAGACTTCGTGGATGTTGAGAAGATTAAAGTCAAGTTCATGACCATGCAGAAAATGTACTCGCCTGA AAAGAAGGTCATGCTGCTGCTGAGAGTTTGCAAATTGATTTACACAGTTATGGAGAATAACTCAGGGAGGC TGTATGGAGCTGATGACTTCTTGCCTGTATTGACATATGTAATAGCTCAATGTGACATGCTGGAGCTGGATA CTGAAATTGAGTACATGATGGAATTGCTGGATCCATCTTTGCTGCATGGAGAAGGAGGCTATTACTTGACGA GCGCTTATGGAGCACTTTCGCTGATCAAGAATTTCCAGGAAGAACAAGCTGCCAGACTGCTAAGTTCAGAA GCCAGAGATACTCTCAGGCAATGGCACAAAAGGAGGACGACTAACAGGACGATACCTTCAGTTGATGATTT TCAGAACTACCTTCGAGTTGCATTCCAGGACGTCAACAGTGGATGCACAGGAAAGACCTTACTAGTAAGAC CGTATATCACTACAGAAGATGTATGCCAGCTGTGTGCTGAGAAGTTTAAAGTGGACAACCCAAAAGAATAT AGCCTCTTTCTTTTTGTTGATGACACCTGGCAGCAACTGACGGAAGATACCTACCCACAGAAGATTAAGGCT GAACTGCACAGCCGTCCGCAGCCCCAGGTCTTCCACTTTGTCTACAAGCGCATTAACAGTGATCCTTACAGT GCCATTTTTCAAAACGACGACTCTGCCTCTTAA |
